# Renal Function Impairment and Associated Factors among HAART Naïve and Experienced Adult HIV Positive Individuals in Southwest Ethiopia: A Comparative Cross Sectional Study

**DOI:** 10.1371/journal.pone.0161180

**Published:** 2016-08-18

**Authors:** Yewulsew Mekuria, Daniel Yilma, Zeleke Mekonnen, Tesfaye Kassa, Lealem Gedefaw

**Affiliations:** 1 Department of Clinical Laboratory Science, Jimma University Specialized Hospital, Jimma, Ethiopia; 2 Department of Internal Medicine, College of Health Sciences, Jimma University, Jimma, Ethiopia; 3 Department of Medical Laboratory Science and Pathology, College of Health Sciences, Jimma University, Jimma, Ethiopia; University of New South Wales, AUSTRALIA

## Abstract

**Background:**

Human immunodeficiency virus (HIV) infection and its treatment cause renal diseases. Renal disease is associated with an increasing cause of morbidity and mortality in HIV positive individuals than in the general population. It has been also associated with adverse outcomes, such as complications of decreased renal functions and progression to renal failure.

**Objective:**

To determine the prevalence and factors associated with renal function impairment among highly active antiretroviral therapy (HAART) naive and HAART experienced adult HIV positive individuals.

**Methods:**

A facility based comparative cross-sectional study was conducted in Jimma University Specialized Hospital (JUSH) from June to September 2014. HIV positive individuals who visited JUSH during the study period were included in the study. Sociodemographic and clinical data were collected using a structured questionnaire. Blood specimen was analyzed for renal function tests. Descriptive statistics, Mann-Whitney U test and logistic regression analysis were done using SPSS version 16 software.

**Results:**

A total of 446 HIV positive individuals, 223 HAART naïve and 223 HAART experienced, were recruited. The overall prevalence of renal function impairment was 18.2% [95%CI: 14.6–21.7]. The prevalence of renal impairment in HAART naive and HAART experienced persons was 28.7% [95%CI: 23.1–34.4] and 7.6% [95%CI: 4.6–11.6], respectively. Age ≥ 50 years (AOR = 3.6; 95% CI 1.4, 9.6), advanced WHO stage (AOR = 2.3; 95% CI 1.1, 4.7), and CD4 count <200 (AOR = 6.9; 95% CI 3.3, 14.2) were independent risk factors among HAART naive participants. Female gender (AOR = 6.6; 95 CI % 1.2, 34), age ≥ 50 years (AOR = 12.1; 95% CI 1.7, 84) and CD4 count <200 (AOR = 17; 95% CI 5.2, 58) were independent risk factors among HAART experienced participants.

**Conclusion:**

The prevalence of renal function impairment was higher among HAART naïve than HAART experienced HIV positive individuals. Renal function impairment was associated with disease advancement and old age.

## Introduction

Human Immunodeficiency virus (HIV) affects every organ system in the body by direct damage or by rendering the host susceptible to opportunistic infections [1]. The commonest sites of infection are lung, brain, heart, gut, kidney, skin, and lymphoid tissues [2]. However, kidney involvement is seen frequently during the course of HIV infection and it has become fourth leading condition contributing to death among those who have progressed to acquired immunodeficiency syndrome (AIDS) patients after sepsis, pneumonia, and liver disease [3]. This can result from common complications of the disease like dehydration and sepsis or effects of nephrotoxic drugs commonly used in the management of AIDS [4]. Renal disease can also be caused by direct role of the HIV infection on the kidney resulting in a distinct entity termed HIV-associated nephropathy (HIVAN), HIV-associated immune complex kidney disease (HIVICK), or thrombotic microangiopathy (TMA) [5].

Renal disease is more common and an increasing cause of morbidity and mortality in HIV positive individuals than in the general population [6–9]. Estimate indicates that renal disease can be found in 30% of HIV positive individuals [10]. The introduction of highly active antiretroviral therapy (HAART) has led to a marked reduction in HIV/AIDS related morbidity and mortality with significant change in the epidemiology of renal disease in HIV positive individuals with a substantial reduction in the incidence of HIVAN [7, 11].

Despite the fact that HAART has decreased HIV associated renal diseases, long-term administration of some HAART has been linked with increased risk of progression to end stage renal diseases (ESRD) [12].

Though regular laboratory monitoring may not be necessary for making sound antiretroviral (ARV) treatment decisions in resource-limited settings [13], the risk of undiagnosed HIV associated renal impairments is worrisome in these settings where routine laboratory testing is often not available [14]. Even in those routine laboratories where tests are available, there is lack of accuracy and precision in analytical measurements including renal function tests [15, 16]. In addition, limited data are available regarding the renal function impairment and its associated factors among HAART naïve and HAART experienced HIV positive individuals in Ethiopia.

Therefore, this study was aimed to determine the prevalence of renal function impairment and its associated factors among HAART naïve and HAART experienced adult HIV positive individuals in Southwest Ethiopia.

## Materials and Methods

### Study design and study population

A facility based comparative cross-sectional study was conducted from June to September 2014, at comprehensive and chronic care center of the Jimma University Specialized Hospital (JUSH) in Jimma Town, Southwest Ethiopia. It is the only teaching and referral hospital in the Southwestern part of the country, providing services for the catchment population of about 15 million people. HAART service is provided at the comprehensive chronic care center.

The sample size was determined using double population proportion general formula by taking two different proportions of renal function impairment [30.1%] for HAART naïve and [12.9%] for HAART experienced participants [17] from the work done in Northwest of Ethiopia. Taking a critical value at 95% confidence level, level of significance 0.05, Power (1- β) = 90%, for the source population of 5, 620, the total sample size calculated was 446. Then we used equal proportion (1:1) of 223 HAART naïve and 223 HAART experienced HIV positive individuals. Accordingly, a total of 446 study participants were approached and consented to be investigated. All outpatient adult HIV positive individuals who had regular follow up and were available during the study period were included in the study, consecutively until the determined sample size was reached in both arms. Pregnant women, participants > 65 years, amputees, known kidney diseases/on treatment were excluded from the study.

### Data collection and analysis

Prior to data collection the data collectors were trained by the principal investigator. Sociodemographic and related clinical data were collected using a structured questionnaire by trained clinical nurses. Blood specimens were collected from each study participants for laboratory investigations. Eight milliliter of venous blood, 4ml with anticoagulant (EDTA, Ethylene diamine tetra acetic acid) and 4ml without anticoagulant with SST test tubes, was collected from each participant. EDTA anticoagulated blood was transported to JUSH laboratory for automated analysis of CD4/CD3 cells/mm^3^ using a BD FACS count machine (Becton Dickinson, San Jose, CA, USA). Random blood glucose was measured using Senso card (Elektronika, Budapest, Hungary). Serum from SST tubes without anticoagulant was analyzed for creatinine using Humastar 80 chemistry analyzer (HUMAN Gesellschaft für Biochemica und Diagnostica mbH, Wiesbaden, Germany).

Serum creatinine was measured by the kinetic alkaline picrate method (Human Diagnostics, Germany) with calibration traceable to reference material NIST SRM 909B level 2. Creatinine clearance was calculated as an estimate of renal function (eGFR) using Cockcroft -Gault equation, which takes into consideration serum creatinine, age, gender, and weight. Renal impairment was classified according to the National Kidney Foundation clinical practice guideline based on the GFR as determined by Cockcroft-Gault method. Accordingly, estimated GFR values ≥ 90ml/min/1.73m^2^, 60–89 ml/min/1.73m^2^, 30–59 ml/min/1.73m^2^,15-29ml/min/1.73 m^2^ and < 15 ml/min/1.73m^2^ was interpreted as normal, mild, moderate, severe and kidney failure. Renal function impairment was defined as eGFR < 60 ml/min [18]. For better quality of the laboratory results, blood specimens were collected, processed and analyzed following standard operating procedures (SOP). Quality control samples were used for all laboratory tests. The results of all laboratory tests were recorded on well standardized report format carefully and attached to questionnaire according to subject’s unique identification number. JUSH Laboratory regularly participates in an external quality assessment program coordinated at national level by the Ethiopian Public Health Institute (EPHI) three to four times per annum.

Data from questionnaires and from laboratory investigation were cleaned and entered to SPSS version 16 software. Simple frequencies (mean, median, and proportions), Mann-Whitney U test, and multiple logistic regression analysis were done using SPSS V-16.0. First the independent variables listed in the table were tested in bivariate logistic regression analysis one by one and then those variables with p-value <0.25 were considered in multivariate logistic regression analysis. Finally, those variables with P-value of ≤ 0.05 in multivariate logistic regression analysis were considered as statistically significant.

### Ethical clearance

Ethical clearance and an approval letter for the study was obtained from Jimma University, College of Health Science Ethical Review Committee to conduct the study at JUSH. A support letter from health science research coordinating office was written to the JUSH. Permission to conduct the study was obtained from the medical director’s office of JUSH. Written informed consent was obtained from the study participants after describing the benefits and risks of the study. Any information concerning the participants was kept confidential and the specimens collected from the participants were analyzed only for the intended purposes. The abnormal study results were communicated to the clinician working in the hospital for appropriate management of individual cases.

## Result

### Socio demographic characteristics of the study participants

A total of 446 (223 HAART naïve and 223 HAART experienced) adult HIV positive individuals who had regular follow up at JUSH comprehensive chronic care center from June to September 2014 participated in the study. Two hundred eighty one (63%) were females. The mean (±SD) age of the study participants was 38.25 (±10.8) years and 35.14 (±9.2) years for HAART naïve and HAART experienced, respectively. Most of the study participants (43.5%) were in the age range of 30–41 years. The mean (±SD) body mass index (BMI) of the study participants was 20.7 (±3.2) and 21.6 (±3.5) for HAART naïve and HAART experienced individuals, respectively; (p = 0.0008).

### Clinical findings of the study participants

Sixty seven (15.02%) of the study participants were in advanced WHO clinical stage (Stage III IV). History of hypertension was reported in 19 (3.36%) of the study participants. The most widely used antiretroviral treatment (ART) regimen in this study was TDF/3TC/EFV in 107 (47.98%) cases followed by AZT/3TC/NVP, 61(27.35%); AZT/3TC/EFV, 20(8.97%); TDF/3TC/NVP, 28(12.56%); ABC/DDI/LPV/r, 4(1.79%); and TDF/DDI/LPV/r 3(1.35%). There was no difference between the CD4 counts (p = 0.817) and glucose level (p = 0.420) of HAART naive and HAART experienced participants (Table 1).

**Table 1:** Comparison of the median of laboratory tests using Mann-Whitney U test of adult HIV positive individuals stratified by HAART status at JUSH. South West Ethiopia, 2014.

Variable	HAART naïve		HAART experienced		P- value
	Median	Interquartile range	Median	Interquartile range	
Creatinine (mg/dl)	0.81	0.29	0.80	0.21	0.287
GFR(CrCl)	88.00	49.00	91.00	36.00	0.011
Glucose (mg/dl)	88.00	24.00	89.00	30.00	0.420
CD4 count (cells/μl)	419.00	449.00	417.00	250.00	0.817

**Note:** GFR, Glomerular filtration rate; CD, cluster of differentiation

### Prevalence of renal function impairment among adult HIV positive individuals

The median of serum creatinine of HAART naïve and HAART experienced study participants was 0.81mg/dl and 0.80 mg/dl, respectively (p = 0.287). The median of creatinine clearance in HAART naive study participants was 88 and the median of serum creatinine clearance level among HAART experienced was 91.00 (p = 0.011) (Table 1).

The overall prevalence of renal function impairment of study participants (eGFR < 60 ml/min) was 18.2% [95%CI: 14.6–21.7] (81/446). The prevalence of renal function impairment among HAART naïve study participant was 28.7% [95%CI: 23.1–34.4] (64/223) whereas among HAART experienced 7.6% [95%CI: 4.6–11.6] (17/223). One hundred fifty (33.6%) of study participants had mild renal function impairment, 58(26%) Vs 92(43.1%) for HAART naïve and HAART experienced study participants, respectively. Seventy four (16.6%) of study participants had moderate renal function impairment, 58 (26%) Vs 16 (7.2%) for HAART naïve and HAART experienced study participants, respectively. Only one (0.4%) of HAART experienced study participants had severe renal function impairment. No renal failure was diagnosed in this study (Table 2).

**Table 2:** Prevalence of renal function impairments stratified by HAART among adult HIV positive individuals at JUSH.

Variables		HAART naive	HAART experienced	Total
		No (%)	No (%)	No (%)
Sex	Male	19(22.6)	2(2.5)	21(12.7)
	Female	45(32.4)	15(10.6)	60(21.4)
Age	≥50	15(48.4)	3(25)	18(41.9)
	<50	46(24.1)	14(6.9)	63(15.6)
BMI	Normal	56(30.6)	16(8.9)	72(19.9)
	Abnormal*	8(20.0)	1(2.3)	9(10.7)
Residence	Urban	51(28.5)	14(8.0)	65(18.3)
	Rural	13(31)	3(6.1)	16(17.6)
Who stage	Advanced	24(47.1)	1(6.2)	25(37.3)
	Non advanced	40(23.3)	16(7.7)	56(14.8)
History of Hypertension	Yes	4(36.4)	1(12.5)	5(26.3)
	No	60(28.3)	16(71.7)	76(17.8)
CD4 count	<200	48(70.6)	5(16.7)	53(54.1)
	≥200	16(10.3)	12(6.2)	28((8.0)
Diabetes status	Diabetes	3(50)	3(13.6)	6(21.4)
	Non diabetes	61(28.1)	14(7)	75(17.5)

NB: *abnormal BMI includes both underweight and overweight.

### Factors independently associated with renal function impairment among adult HIV positive individuals

Multivariate logistic regression analysis showed that being HAART naïve, being female, old age ≥ 50 years, abnormal BMI, advanced WHO stage, and low CD4 count remained independently associated for renal function impairment (Table 3).

**Table 3:** Bivariate and multivariate logistic regression analysis for renal impairment among adult HIV positive individuals at JUSH, Southwest Ethiopia, 2014.

Variable		COR (95%CI)	p-value	AOR (95%CI)	p-value
Sex	Male	1*	0.036	1*	0.006
	Female	1.7(1.00–3.00)		2.4(1.2–4.70)	
Age	≥50	4.3(2.20–8.30)	<0.001	4.4(1.90–10.00)	0.002
	<50	1*		1*	
BMI	Normal	1*	0.033	1*	0.013
	Abnormal*	2.3(1.00–5.00)		3.1(1.10–8.00)	
WHO stage	Non advanced	1*	0.001	1*	0.008
	Advanced stage	4.2(2.50–7.00)		2.4(1.20–4.70)	
History of Hypertension	Yes	1.7(0.60–4.80)	0.321		
	No	1*			
CD4 count	≥200	1*	<0.001	1*	<0.001
	<200	9.9(5.70–17.20)		8.7(4.60–16.30)	
Status of HAART	Naïve	4.6(2.60–8.20)	<0.001	2.7(1.40–5.40)	0.003
	Experienced	1*		1*	
Diabetes status	Diabetes	1.2(0.50–3.20)	0.604		
	Non diabetes	1*			

1* = Reference category, COR = Crud Odds Ratio, AOR = Adjusted Odds Ratio,

*abnormal BMI includes both underweight and overweight.

Old age ≥50 years, advanced WHO stage, and low CD4 count were independently associated factors for renal function impairment among HAART naïve individuals (Table 4). On the other hand, being female, old age ≥50 years and low CD4 count were independently associated factors for renal function impairment among HAART experienced individuals (Table 5).

**Table 4:** Bivariate and multivariate logistic regression analysis for predictors of renal impairment among HAART naive HIV positive individuals participated in the study, south west Ethiopia, 2014.

Variable		COR (95%CI)	p-value	AOR (95%CI)	p-value
Sex	Male	1*	0.181	1*	0.082
	Female	1.5(0.82–2.80)		1.8(0.91–3.90)	
Age	≥50	1*	0.003	1*	0.005
	<50	3.5(1.40–6.70)		3.6(1.40–9.60)	
BMI	Normal	1*	0.114	1*	0.066
	Abnormal*	2.0(0.85–4.80)		2.5(0.90–7.20)	
WHO stage	Non advanced	1*	0.001	1*	0.020
	Advanced	2.8(1.50–5.10)		2.3(1.10–4.70)	
History of Hypertension	Yes	1.5(0.43–5.30)	0.518		
	No	1*			
CD4 count	≥200	1*	<0.001	1*	<0.001
	<200	7(3.60–13.50)		6.9(3.30–14.20)	
Diabetes status	Diabetes	3.1(1.40–6.70)	0.003	4.3(0.66–28.70)	0.134
	Non Diabetes	1*		1*	

1* = Reference category, COR = Crud Odds Ratio, AOR = Adjusted Odds Ratio,

*abnormal BMI includes both underweight and overweight.

**Table 5:** Bivariate and multivariate logistic regression analysis for predictors of in HAART experienced HIV patients involved in the study, south west Ethiopia, 2014.

Variable		COR(95%CI)	p-value	AOR(95%CI)	p-value
Sex	Male	1*	0.044	1*	0.009
	Female	4.6(1.00–20)		6.6(1.2–34)	
Age	≥50	4.6(1.10–19.30)	0.032	12.1(1.70–84)	0.017
	<50	1*		1*	
BMI	Normal	1*	0.168	1*	0.071
	Abnormal*	4.2(0.54–32.70)		7.2(0.49–11)	
WHO stage	Non advanced	1*	<0.001		0.223
	Advanced	2.8(1.50–5.10)		2.4(0.59–9.70)	
History of Hypertension	Yes	1.5(0.43–5.30)	0.518		
	No	1*			
CD4 count	≥200	1*	<0.001	1*	<0.001
	<200	14.36(4.80–42.32)		17(5.2–58)	
Regimen	TDF	1.5(0.57–4.10)	0.388		
	Non TDF	1*			
Diabetes status	Diabetes	2.1(0.56–80)	0.273		
	Non diabetes	1*			

1* = Reference category, COR = Crud Odds Ratio, AOR = Adjusted Odds Ratio,

*abnormal BMI includes both underweight and overweight.

## Discussion

The overall prevalence of renal function impairment in our study based on glomerular filtration rate using the Cockcroft-Gault method was 18.2%. This result was lower as compared to a report from northwest of Ethiopia which showed 21% had renal impairment [17]. However, the prevalence of renal impairment in this study was higher than studies from Burundi (4.9%), Ghana (10.8%), and China (5.6%) [19, 20, 21]. Population variation, study design, sample size, definition used to classify renal impairments, and use of GFR different estimating formulas may contribute to the differences observed.

The prevalence of renal function impairment among HAART naïve individuals in this study was 28.7%. It is comparable to a study conducted in other part of Ethiopia (31.1%), Tanzania (25%), and Nigeria (26%) [17, 14, 22]. However, the prevalence in this study was higher than studies from other African countries; 3% in Cameroon [23], 8% in Zambia [24], 11.5% in Kenya [25], 20% in Uganda [26], 23% in Malawi [27] and lower than reports from Lesotho (35%) [28]. This variation might be due to the difference in inclusion and exclusion criteria of the study participants, sample size and definition of renal impairment used. For instance, in the study conducted in Kenya and Ghana HIV positive individuals with hypertension and diabetes mellitus were excluded, in Malawi participants who had advanced stage of HIV infection were excluded from the study.

The prevalence of renal function impairment among HAART experienced individuals was 7.6% which is comparable with studies reported in Uganda (6%) [26], and in Nigeria (6.9%) [22]. However, the prevalence is higher than studies done in Tanzania (1.1%) [29], and in Lesotho (5.5%) [28], and lower than the prevalence reported from Ethiopia (12.1%) [17], and Ghana (14.5%) [20]. This wide variation could be in part due to differences in HAART regimen, stage of HIV infection and the method used to estimate GFR. Use of modified diet of renal disease formula to estimate GFR in Ghana and the number of study subjects may contribute to the differences observed.

The median creatinine clearance level was significantly higher in HAART experienced study participants than HAART naive study participants. This finding is supported by Getachew et.al [17], Yusuf R et.al [22]. The finding of higher serum creatinine clearance in HAART experienced study participants may be due to the improvement in renal function by HAART drugs.

The prevalence of renal impairment was significantly higher in HAART naïve study participants compared to HAART experienced study participants. HAART naïve HIV positive study participants had 2.7 times more risk of developing renal impairment compared to those HAART experienced study participants. Being HAART naïve study participants were also independently and significantly associated with renal impairment in adult HIV positive study participants. This is also supported by other study conducted in Ethiopia [17]. The finding of lower prevalence of renal function impairment in HAART experienced study participants may indirectly indicate the effectiveness of HAART in reducing HIV related renal disease by reducing the occurrence of HIVAN, opportunistic infections, HIV associated metabolic complication, and by improving the immunologic response of HIV positive individuals. The implementation of the current World Health Organization (WHO) test and treat approach might have a great impact in reducing HIV related renal diseases [30].

Renal function is known to decline with age. Older age is an established risk factor for a decline in creatinine clearance in the general population [31]. According to this study the observed renal impairment in HIV positive individuals with old age ≥ 50 years was found to be 4.4 times more than those less than 50 years age. Similarly, older age has been independently associated with renal function decline among HIV-positive individuals in other studies [28, 32, 33].

This study showed that renal impairment among females was 2.4 times more than male study participants. Female gender was independently and significantly associated with renal impairment in this study. This is in agreement with the findings of studies conducted in France [33], and U.S.A [32]. This might be due to the higher proportion women make up the majority of total number people living with HIV/AIDS in Ethiopia and in this study.

Advanced WHO stage, old age, and CD4 count <200 cells/mm^3^ were identified as the predictors of renal impairment in HAART naive participants. On the other hand, female gender, old age, and low CD4 count were found to be the predictors of renal impairment in HAART experienced study participants.

In general, this study was a comparative cross sectional study which tried to identify independent factors of renal function impairment for HAART naïve and HAART experienced adult HIV positive individuals. The limitations of the study are: first of all creatinine was measured at a single point in time; therefore, it may have included short term, reversible causes of renal impairment which may overestimate renal impairment. Secondly, use of the Cockcroft-Gault formula to assess renal function in HIV individuals may not be accurate enough to allow a firm conclusion of renal function impairment and can lead to misclassification of some patients. Thirdly, this study is limited by its cross sectional design, not longitudinal, preventing assessment of whether risk factors caused or resulted from decreased renal function. However, being prospective study, use of large sample size, assessment of associated factors for renal function impairment could be considered as the strength of this study.

## Conclusion

The overall renal function impairment was 18.2%. Renal function impairment was higher among HAART naïve than HAART experienced study participants. Female gender, old age, low CD4 count, being HAART naïve, advanced WHO clinical stage and abnormal BMI were associated with renal function impairments in the study participants. For HAART naïve study participants, age ≥50 years, advanced WHO stage, and low CD4 were risk factors for renal function impairment. However old age, female gender and low CD4 count were risk factors for HAART experienced HIV positive individuals. This study shows that HAART initiation might have important role in reducing HIV related renal diseases and improving renal function.
